# Preoperative Axillary Nodal Burden in Early Breast Cancer: Assessment With Ultrasound-Guided Fine-Needle Aspiration Cytology and Sentinel Lymph Node Biopsy

**DOI:** 10.7759/cureus.113339

**Published:** 2026-07-25

**Authors:** Mitu Debnath, Hasnat Zaman Zim, S. M. Syeed-Ul-Alam, Hasan Shahrear Ahmed

**Affiliations:** 1 Department of Surgery, Sher-E-Bangla Medical College Hospital, Barisal, BGD; 2 Department of General Surgery, Bangladesh Medical University (BMU), Dhaka, BGD; 3 Department of Surgical Oncology, Bangladesh Medical University (BMU), Dhaka, BGD

**Keywords:** axillary lymph nodes, axillary nodal burden, early breast cancer, fine needle aspiration for cytology, sentinel lymph node biopsy

## Abstract

Background

Axillary assessment is a key component of breast cancer care because nodal involvement affects prognosis, staging, and treatment selection. Ultrasound-guided fine-needle aspiration cytology (US-FNAC) can provide preoperative cytological evidence of axillary metastasis. This study assessed whether positive US-FNAC findings in clinically node-negative (cN0) early breast cancer were associated with a higher axillary nodal burden than sentinel lymph node biopsy (SLNB) positivity.

Methods

This prospective observational study was performed at Bangladesh Medical University (BMU), Dhaka, Bangladesh, from August 2021 to July 2022. The study included 32 women with histologically confirmed early breast cancer and a cN0 axilla. All patients underwent preoperative axillary ultrasonography (AUS). Patients with detectable axillary lymph nodes (ALNs) underwent US-FNAC; those with positive US-FNAC results proceeded directly to axillary lymph node dissection (ALND), whereas those with negative US-FNAC results or no detectable lymph nodes underwent SLNB, followed by ALND if SLNB was positive. Final axillary histopathology served as the reference standard for evaluating the diagnostic performance of US-FNAC and comparing axillary nodal burden.

Results

AUS showed no lymph nodes in 16 (50.0%) patients, suspicious nodes in six (18.8%) patients, and non-suspicious nodes in 10 (31.2%) patients. The median metastatic ALN count was higher among US-FNAC-positive patients than among SLNB-positive patients (4.0 vs. 2.5; p = 0.094). More than three metastatic nodes were found in six (85.7%) US-FNAC-positive patients and three (37.5%) SLNB-positive patients. US-FNAC had a sensitivity of 87.5%, specificity of 100.0%, positive predictive value (PPV) of 100.0%, negative predictive value (NPV) of 88.9%, and an overall accuracy of 93.7%.

Conclusion

In patients with early breast cancer and a cN0 axilla, positive preoperative US-FNAC was associated with heavier axillary metastatic involvement. These findings support the role of US-FNAC in preoperative axillary staging and surgical decision-making.

## Introduction

Information on axillary lymph node (ALN) involvement remains fundamental in primary breast cancer, as nodal disease helps determine prognosis and treatment planning and is closely linked to overall and disease-free survival [1]. Axillary surgery for early breast cancer has progressively shifted toward less invasive management, aiming to preserve oncological safety while reducing procedure-related morbidity [2]. Even so, reliable preoperative assessment of the axilla is still difficult because physical examination may fail to detect metastatic nodes in many patients who appear clinically node-negative (cN0) [3].

Assessment of the axilla is therefore important for staging, prognostic classification, and selection of local or systemic treatment [4]. Axillary lymph node dissection (ALND) was previously the routine method for nodal staging, but sentinel lymph node biopsy (SLNB) is now preferred for cN0 early breast cancer because it stages the axilla accurately with less morbidity than ALND [5]. The ACOSOG Z0011 trial further reshaped practice by showing that selected women with T1-T2 tumours and one or two positive sentinel nodes, treated with breast-conserving surgery, whole-breast radiotherapy, and systemic therapy, can safely avoid completion ALND [6].

Despite the move toward conservative axillary treatment, preoperative recognition of a high nodal burden remains clinically useful. Patients with extensive nodal disease may be candidates for ALND or neoadjuvant systemic treatment, whereas those with limited nodal disease may receive unnecessary axillary surgery if treatment is based only on a positive preoperative needle biopsy [2,7]. Axillary ultrasonography (AUS) is widely used before surgery to examine regional lymph nodes. If a suspicious node is seen, ultrasound-guided fine-needle aspiration cytology (US-FNAC) or core needle biopsy can confirm metastasis before definitive surgery [4,7].

Prior studies indicate that needle biopsy added to AUS increases specificity and positive predictive value (PPV) for nodal metastasis and can reduce unnecessary SLNB in selected patients [4,8]. In the post-Z0011 setting, however, interpretation of a positive preoperative axillary biopsy is more nuanced because some patients with biopsy-proven metastasis still have only limited nodal disease and may not require ALND [2,9,10]. Additional evaluation is therefore needed to clarify whether US-FNAC can identify patients who are more likely to have a substantial axillary metastatic burden.

This study assessed the performance of US-FNAC for detecting axillary lymph node metastasis and compared axillary nodal burden between US-FNAC-positive and SLNB-positive patients with early breast cancer and a cN0 axilla.

## Materials and methods

Study design and participants

This prospective observational study was carried out in the Department of General Surgery, Bangladesh Medical University (BMU), Dhaka, Bangladesh, between August 2021 and July 2022. Consecutive female patients attending the outpatient and inpatient services were screened. The final study cohort included 32 women with histopathologically confirmed primary invasive early breast cancer and a cN0 ipsilateral axilla.

Sample size was estimated with Buderer's method for studies evaluating diagnostic test performance. The calculation used an expected US-FNAC specificity of 95.7%, a 95% confidence level, 10% absolute precision, and an assumed disease prevalence of 50%. This produced a required sample of 31.6 patients, so 32 participants were enrolled.

Eligible participants were women aged 18 years or above with histologically confirmed primary invasive early breast cancer, clinical T1-T3 disease, and a cN0 ipsilateral axilla. We did not include patients with prior axillary surgery, recurrent breast cancer, neoadjuvant chemotherapy exposure, bilateral disease, another malignancy, distant metastasis, breast tuberculosis, mastitis, upper limb infection, or malignant disease involving the upper limb.

Study procedure

**Figure 1:**
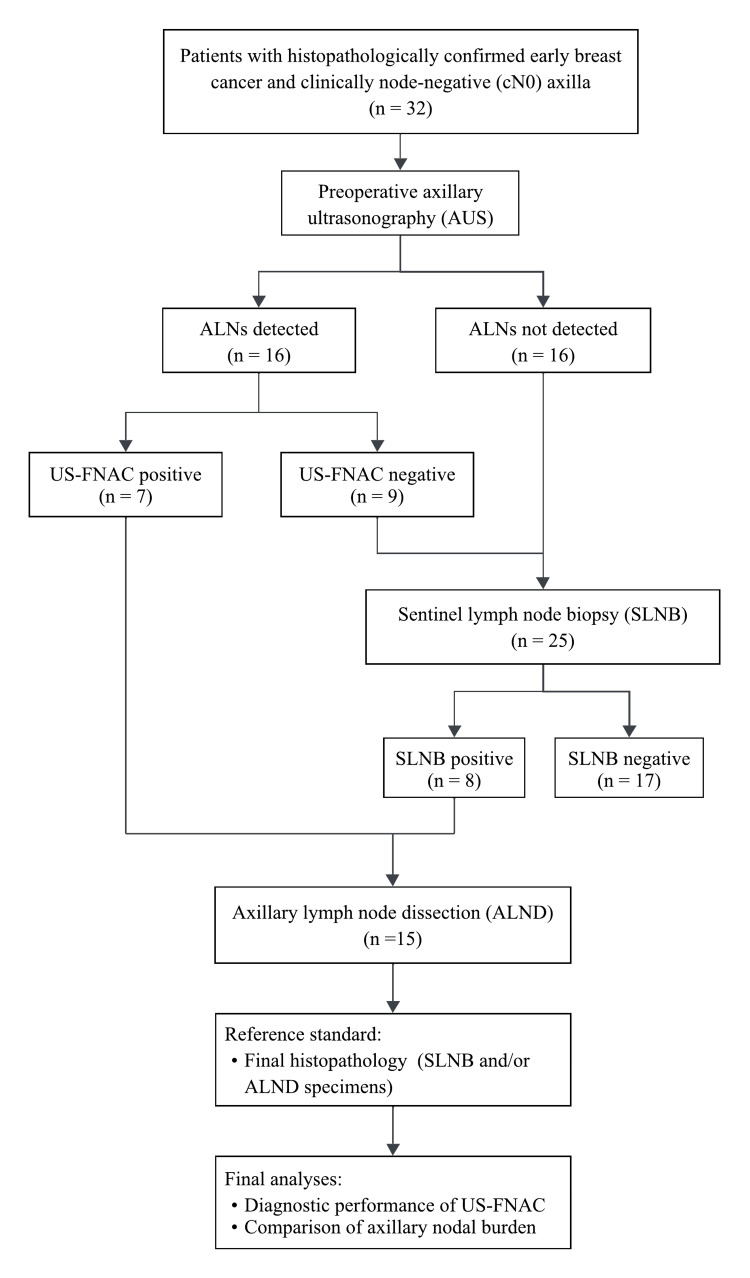
Participant flow and axillary staging pathway ALNs: axillary lymph nodes; US-FNAC: ultrasound-guided fine-needle aspiration cytology; SLNB: sentinel lymph node biopsy

All participants underwent preoperative AUS. Patients with detectable ALNs underwent US-FNAC. Patients with positive US-FNAC results proceeded directly to ALND, whereas those with negative US-FNAC results subsequently underwent SLNB. Patients without detectable ALNs on AUS also underwent SLNB. Patients with metastatic sentinel lymph nodes subsequently underwent ALND. Final axillary histopathology obtained from SLNB and/or ALND specimens served as the reference standard. Detectable ALNs were also assessed for sonographic features suggestive of metastatic involvement and were classified as suspicious lymph nodes in the presence of one or more of the following features: cortical thickening (≥3 mm), focal or eccentric cortical thickening, partial or complete loss of the fatty hilum, a rounded nodal configuration (low longitudinal-to-transverse axis ratio), nodal enlargement, or irregular cortical morphology. The participant flow and diagnostic pathway are illustrated in Figure 1.

Sentinel lymph node biopsy (SLNB)

Sentinel node mapping was performed with methylene blue dye. Three to four millilitres of dye were injected into the subareolar region of the affected breast, after which stained sentinel nodes were identified and removed. Patients with metastatic sentinel nodes underwent completion ALND. For these patients, the total ALN count was obtained by adding the sentinel nodes removed during SLNB to the non-sentinel nodes removed during ALND.

Data collection

Study data were recorded prospectively on a structured collection form (Appendix 1). The recorded variables were age, tumour site and size, AUS findings, US-FNAC results, SLNB and ALND findings, histopathological tumour type, total ALN count, and metastatic ALN count. These variables were used to describe the cohort, evaluate US-FNAC performance, and compare axillary nodal burden.

Outcome measures and statistical analysis

The main outcome was the ability of US-FNAC to detect ALN metastasis in patients with early breast cancer and a cN0 axilla. A secondary outcome was the difference in axillary nodal burden between the US-FNAC-positive and SLNB-positive groups.

Final axillary nodal status on histopathological examination of lymph nodes obtained through SLNB and/or ALND was used as the reference standard. Sensitivity, specificity, PPV, negative predictive value (NPV), and overall accuracy were calculated with 95% confidence intervals (CIs). Continuous data were summarized as mean ± standard deviation (SD) or median with interquartile range, according to the data distribution. Categorical data were reported as n (%). Fisher's exact test or the chi-square test was used for categorical comparisons, as appropriate, and the Mann-Whitney U test was used for continuous variables. Diagnostic indices were derived from 2 × 2 contingency tables, and exact Clopper-Pearson CIs were used. Analyses were performed in IBM SPSS Statistics for Windows, Version 26 (Released 2018; IBM Corp., Armonk, NY, USA), with p < 0.05 considered statistically significant.

## Results

The study included 32 women with histopathologically confirmed primary invasive early breast cancer and a cN0 axilla. The mean age was 47.9 ± 10.5 years, and 21 (65.6%) patients were aged 38-57 years. Left-sided disease was present in 19 (59.4%) patients and right-sided disease in 13 (40.6%) patients. The upper outer quadrant was the most frequent tumour site, seen in 12 (37.5%) patients. Most tumours were clinically T2 in 23 (71.9%) patients, while seven (21.9%) were T1 and two (6.2%) were T3. Invasive ductal carcinoma was the predominant histological type in 28 (87.5%) patients, and Grade II was the most common histological grade in 25 (78.1%) patients. Most tumours were estrogen receptor (ER)-positive in 21 (65.6%) patients, progesterone receptor (PR)-positive in 19 (59.4%) patients, and human epidermal growth factor receptor 2 (HER2)-negative in 27 (84.4%) patients. Baseline clinicopathological characteristics are presented in Table 1.

**Table 1:** Clinicopathological profile of the study cohort (n = 32) T: clinical tumour stage (TNM classification); ER: estrogen receptor; PR: progesterone receptor; HER2: human epidermal growth factor receptor 2

Variable	n (%)
Age group (years)	
≤37	6 (18.8)
38-57	21 (65.6)
≥58	5 (15.6)
Mean age, years (mean ± SD)	47.9 ± 10.5
Body mass index (BMI)	
Normal weight	10 (31.2)
Overweight/Obese	22 (68.8)
Side of breast involvement	
Right	13 (40.6)
Left	19 (59.4)
Tumour location	
Upper outer quadrant	12 (37.5)
Upper inner quadrant	10 (31.3)
Lower outer quadrant	5 (15.6)
Lower inner quadrant	3 (9.4)
Central	2 (6.2)
Clinical T stage	
T1	7 (21.9)
T2	23 (71.9)
T3	2 (6.2)
Histological type	
Invasive ductal carcinoma	28 (87.5)
Invasive lobular carcinoma	1 (3.1)
Others	3 (9.4)
Histological grade	
Grade I	3 (9.4)
Grade II	25 (78.1)
Grade III	4 (12.5)
ER status	
Positive	21 (65.6)
Negative	11 (34.4)
PR status	
Positive	19 (59.4)
Negative	13 (40.6)
HER2 status	
Positive	5 (15.6)
Negative	27 (84.4)

Preoperative AUS detected ALNs in 16 (50.0%) patients, while no ALN was visualized in 16 (50.0%) patients. Among patients with detected ALNs, six (37.5%) had suspicious nodes, and 10 (62.5%) had non-suspicious nodes. When calculated against the full cohort, these corresponded to six (18.8%) and 10 (31.2%) patients, respectively. Suspicious nodes were larger than non-suspicious nodes, with median sizes of 1.50 cm (IQR, 1.10-1.70) and 0.72 cm (IQR, 0.54-1.10), respectively (U = 7.00, p = 0.010). Table 2 summarizes the sonographic findings among patients with detected ALNs.

**Table 2:** Sonographic profile of detected axillary lymph nodes (n = 16) Percentages are based on patients with detected axillary lymph nodes (n = 16). The U statistic and p-value for lymph node size were obtained using the Mann-Whitney U test. IQR: interquartile range

Characteristic	Suspicious nodes (n = 6)	Non-suspicious nodes (n = 10)	Statistic	p-value
Detected nodes, n (%)	6 (37.5)	10 (62.5)	-	-
Node size, median (IQR), cm	1.50 (1.10-1.70)	0.72 (0.54-1.10)	U = 7.00	0.010

Of the 16 patients with ALNs visible on ultrasound, seven (43.8%) had positive US-FNAC results, and nine (56.2%) had negative US-FNAC results. Positive cytology was found in five of six (83.3%) patients with suspicious lymph nodes and in two of 10 (20.0%) patients with non-suspicious lymph nodes. The cross-tabulation of AUS category and US-FNAC results is shown in Table 3.

**Table 3:** Axillary ultrasound category by US-FNAC result (n = 16) Percentages are calculated within each row. AUS: axillary ultrasonography; US-FNAC: ultrasound-guided fine-needle aspiration cytology

AUS category	US-FNAC positive n (%)	US-FNAC negative n (%)	Total
Suspicious nodes	5 (83.3)	1 (16.7)	6
Non-suspicious nodes	2 (20.0)	8 (80.0)	10
Total	7 (43.8)	9 (56.2)	16

ALND was performed in 15 patients after either positive US-FNAC or positive SLNB. Histopathology showed a heavier metastatic nodal burden in the US-FNAC-positive group than in the SLNB-positive group. The median number of metastatic ALNs was 4.0 (IQR, 4.0-14.0) for US-FNAC-positive patients and 2.5 (IQR, 1.2-5.5) for SLNB-positive patients (U = 13.00, p = 0.094). More than three metastatic ALNs were identified in six of seven (85.7%) US-FNAC-positive patients and three of eight (37.5%) SLNB-positive patients (OR = 0.10, p = 0.119). Total and metastatic ALN counts were assessed from SLNB and/or ALND pathology, and the group comparison is provided in Table 4.

**Table 4:** Nodal burden among patients undergoing axillary lymph node dissection after positive US-FNAC or positive SLNB (n = 15) ^a^ Mann-Whitney U test, U statistic reported; ^b^ Fisher's exact test, odds ratio reported as the test statistic. ALNs: axillary lymph nodes; US-FNAC: ultrasound-guided fine needle aspiration cytology; SLNB: sentinel lymph node biopsy

Variable	US-FNAC+ (n = 7)	SLNB+ (n = 8)	Statistic	p-value
Metastatic ALN count, median (IQR)	4.0 (4.0-14.0)	2.5 (1.2-5.5)	U = 13.00	0.094^a^
Metastatic ALN category, n (%)				
≤3 positive nodes	1 (14.3)	5 (62.5)	OR = 0.10	0.119^b^
>3 positive nodes	6 (85.7)	3 (37.5)		

Using final histopathological examination of SLNB and/or ALND specimens as the reference standard, US-FNAC showed 87.5% sensitivity (95% CI, 47.3-99.7), 100.0% specificity (95% CI, 63.1-100.0), 100.0% PPV (95% CI, 59.0-100.0), 88.9% NPV (95% CI, 51.8-99.7), and 93.7% overall accuracy (95% CI, 69.8-99.8) for ALN metastasis. Table 5 presents these diagnostic performance estimates.

**Table 5:** Diagnostic performance of ultrasound-guided fine needle aspiration cytology (US-FNAC) using final axillary pathology as the reference standard Final histopathological examination of axillary lymph nodes obtained through sentinel lymph node biopsy and/or axillary lymph node dissection was used as the reference standard. Confidence intervals were calculated with the exact Clopper-Pearson method.

Diagnostic parameter	Estimate (%)	95% CI
Sensitivity	87.5	47.3-99.7
Specificity	100.0	63.1-100.0
Positive predictive value (PPV)	100.0	59.0-100.0
Negative predictive value (NPV)	88.9	51.8-99.7
Overall accuracy	93.7	69.8-99.8

## Discussion

This study evaluated US-FNAC within the preoperative axillary work-up of women with early breast cancer and a cN0 axilla. The findings indicate that US-FNAC performed well for identifying axillary metastasis and that positive cytology was associated with a higher metastatic nodal burden than positive SLNB. Therefore, US-FNAC may be useful not only for confirming nodal disease before surgery but also for identifying patients who are more likely to have extensive axillary involvement.

Preoperative AUS can reveal nodal abnormalities that are not detected clinically, which explains its frequent use in breast cancer assessment. In this study, suspicious ALNs were significantly larger than non-suspicious ALNs, consistent with earlier work linking cortical thickening, loss of the fatty hilum, nodal enlargement, and rounded nodal morphology with metastatic disease [11]. The high proportion of positive cytology among suspicious nodes also supports the use of ultrasound to select patients for preoperative needle sampling.

With final pathology used as the reference standard, US-FNAC produced high values for sensitivity, specificity, PPV, NPV, and overall accuracy. These results are comparable with published evidence [12]. Castellano et al. reported a sensitivity of 83.7%, a specificity of 100%, a PPV of 100%, and an NPV of 74.6%, while Park et al. also found very high specificity and PPV for US-FNAC [4,9]. Houssami et al., in a meta-analysis, reported pooled sensitivity and specificity of approximately 80% and 98%, respectively [13]. Across studies, the consistently high specificity indicates that positive US-FNAC is a reliable sign of axillary metastasis and can assist preoperative planning.

Another aim was to examine whether US-FNAC positivity identified patients with greater axillary disease than SLNB positivity. The US-FNAC-positive group had a higher median metastatic node count, and most patients in this group had more than three metastatic ALNs. By comparison, nodal involvement in the SLNB-positive group was generally lower. Although statistical significance was not reached, most likely because of the small sample size, the magnitude of the observed difference suggests clinical relevance. These findings support the view that US-FNAC tends to detect patients with more extensive axillary disease rather than simply identifying any nodal metastasis.

Our observations agree with previous studies. Castellano et al. reported that patients with positive US-FNAC more often had more than three metastatic nodes than patients with positive SLNB [9]. Liang et al. also found a larger nodal burden among patients with positive preoperative node biopsy than among those detected by SLNB [14]. Lloyd et al. and Cipolla et al. similarly reported more positive nodes at ALND among patients whose nodal disease was proven by ultrasound-guided needle biopsy than among those with positive sentinel nodes [3,15]. Together, these findings suggest that preoperative US-FNAC preferentially identifies patients with a heavier axillary tumour burden.

These results should be interpreted in light of modern axillary management. Since ACOSOG Z0011, routine ALND has been avoided in selected patients with limited sentinel node disease, but patients with a high nodal burden still need to be recognized before treatment [2,6]. Such patients may require broader axillary treatment or neoadjuvant systemic therapy. The present findings suggest that US-FNAC can contribute to this preoperative risk stratification by identifying patients with a higher probability of extensive nodal involvement.

In clinical practice, a positive preoperative US-FNAC result may help the surgical team plan axillary management and may reduce the need for an additional staging procedure in selected patients. This is especially relevant in resource-limited settings, where SLNB may be less available or less affordable. Combining AUS with US-FNAC may provide a practical approach to preoperative axillary staging, while early recognition of a high nodal burden can support multidisciplinary planning and reduce repeated axillary procedures.

The strengths of this study include prospective enrolment, histopathological confirmation of invasive breast cancer, inclusion of cN0 patients, use of a consistent diagnostic pathway, and final pathology as the reference for nodal assessment. The study also compared nodal burden between US-FNAC-positive and SLNB-positive patients, an issue that remains relevant after ACOSOG Z0011 [2,14,16]. The limitations should also be noted. It was conducted at a single centre with a relatively small sample size, resulting in limited statistical power and wide confidence intervals for some diagnostic estimates. The relatively small number of outcome events precluded multivariable regression analysis; therefore, the findings represent unadjusted associations. The two comparison groups arose from different stages of the predefined diagnostic pathway rather than from independent allocation, introducing potential selection (spectrum) bias in comparisons of axillary nodal burden. AUS and US-FNAC are operator-dependent procedures, and formal blinding between imaging, cytological, and histopathological assessments was not implemented, introducing the potential for observer bias. Furthermore, although clinicopathological variables such as tumour biology, receptor status, histological grade, and tumour stage may influence nodal metastasis, the present study was not designed or adequately powered to evaluate these associations independently. Finally, the applicability of US-FNAC is limited to patients with detectable ALNs on ultrasonography, as patients without detectable nodes were managed directly with SLNB according to the study protocol. Larger multicentre prospective diagnostic accuracy studies are needed to validate these findings and further define the role of US-FNAC in contemporary axillary staging.

## Conclusions

In this cohort of early breast cancer patients with a cN0 axilla, US-FNAC showed high specificity and strong overall performance for detecting ALN metastasis. Positive preoperative US-FNAC was associated with greater axillary nodal burden than positive SLNB. These findings support the use of US-FNAC as a preoperative triage tool to assist axillary staging and surgical decision-making.

## Appendices

Appendix 1: Structured data collection form

Serial number:

Principal Investigator: Dr. Mitu Debnath

Department: Department of General Surgery, BSMMU

Particulars of patient:

Age:  years

Gender:  Female

Date of Operation:

Name of Procedure: 

Breast conserving surgery (BCS)/Mastectomy

SLNB/ALND/SLNB followed by ALND

Family history:

Breastfeeding history:

Hormonal contraceptive:

Menopausal Status: Premenopausal=1; Postmenopausal=2

Preoperative comorbidities:

  DM=1

  HTN=2

  IHD =3

  COPD/Asthma=4

  Others=5

Body mass index (BMI):

Clinical presentation:

  Breast lump (side, quadrant/location)

  Nipple retraction/discharge

  Others

Clinical signs:

AUS findings:

  Suspicious ALNs=1

  Normal ALN=2

  No ALN=3

  Size of the detected ALN: ______ cm

Cytological findings of US-FNAC:

  Positive = 1

  Negative = 2

  Inadequate = 3

Pathological findings in SLNB-positive patients:

  Number of SLNs removed:

  Number of positive SLNs:

Tumour size (Clinical T stage):

  ≤2 cm (T1) = 1

  >2 to ≤5 cm (T2) = 2

  >5 cm (T3) = 3

Pathological Type:

  Invasive ductal carcinoma (IDC) = 1

  Invasive lobular carcinoma (ILC) = 2

  Others = 3

Multicentricity of the tumour: 1 = Yes; 2 = No

Histopathological grading (According to the Nottingham modification of the Bloom-Richardson grading system):

  Grade I=1

  Grade II=2

  Grade III=3

Biomarker status:

ER                             1=Positive        2=Negative

PR                             1=Positive        2=Negative

HER2                        1=Positive        2=Negative     Equivocal =3

Axillary nodes excised at ALND:

  Number of excised nodes:

  Number of positive nodes:

Signature of the investigator
